# Metal Matrix Composites Synthesized by Laser-Melting Deposition: A Review

**DOI:** 10.3390/ma13112593

**Published:** 2020-06-06

**Authors:** Muhammad Arif Mahmood, Andrei C. Popescu, Ion N. Mihailescu

**Affiliations:** 1 Faculty of Physics, University of Bucharest, Magurele, 077125 Ilfov, Romania; arif.mahmood@inflpr.ro; 2 Laser Department, National Institute for Laser, Plasma and Radiation Physics (INFLPR), Magurele, 077125 Ilfov, Romania; 3 Center for Advanced Laser Technologies (CETAL), National Institute for Laser, Plasma and Radiation Physics (INFLPR), Magurele, 077125 Ilfov, Romania

**Keywords:** 3D printing, laser-melting deposition, wire- and powder-based laser-melting depositions, metal matrix composites, mechanical properties of metal matrix composites

## Abstract

Metal matrix composites (MMCs) present extraordinary characteristics, including high wear resistance, excellent operational properties at elevated temperature, and better chemical inertness as compared to traditional alloys. These properties make them prospective candidates in the fields of aerospace, automotive, heavy goods vehicles, electrical, and biomedical industries. MMCs are challenging to process via traditional manufacturing techniques, requiring high cost and energy. The laser-melting deposition (LMD) has recently been used to manufacture MMCs via rapid prototyping, thus, solving these drawbacks. Besides the benefits mentioned above, the issues such as lower ultimate tensile strength, yield strength, weak bonding between matrix and reinforcements, and cracking are still prevalent in parts produced by LMD. In this article, a detailed analysis is made on the MMCs manufactured via LMD. An illustration is presented on the LMD working principle, its classification, and dependent and independent process parameters. Moreover, a brief comparison between the wire and powder-based LMDs has been summarized. Ex- and in-situ MMCs and their preparation techniques are discussed. Besides this, various matrices available for MMCs manufacturing, properties of MMCs after printing, possible complications and future research directions are reviewed and summarized.

## 1. Introduction

Additive manufacturing, abbreviated as AM, is the reverse of subtractive manufacturing technologies and defined by *ASTM F2792* as “a procedure of joining the materials usually layer by layer to form 3D objects using a computer aided design (CAD) model” [1]. There are various AM processes these days. However, they can be classified into two major categories: direct- and indirect-AM methods [2]. In direct-AM techniques, a laser beam is used as the heat source to melt the powder or wire feedstock. Thus, parts with higher density, purity, and excellent mechanical properties can be produced with less energy and time in comparison to indirect-AM processes [2,3,4,5]. Indirect-AM processes are mainly composed of green bodies and binder materials, which are mixed to manufacture a 3D structure. Usually, the process is followed by a sintering process to eliminate the binder material. In the end, they are densified by conventional manufacturing processes [2]. Table 1 summarizes the different AM processes classified based on direct- and indirect-AM methods.

Every AM process involves the following steps to achieve a 3D printed object [22]:

i. Initially, CAD software is used to build a 3D model, which is to be printed.
ii. This CAD model is converted into stereolithography (STL) format (stereolithography, principally recognized AM practice, implemented as a standard in AM industry). This file is the wedge-shaped illustration of a 3D CAD model.
iii. The file from the step (ii) is sliced into several thin cross-sectional layers using a slicing software. In this step, the building orientation is defined.
iv. Following on, the actual part is printed by a machine using CNC (Computer Numerical Control) codes based on the sliced file. These CNC codes define the smooth, and jerk-free movements of the deposition head, resulting in higher efficiency and better-quality depositions [23,24].
v. In the final step, post-processing steps such as surface treatments, sintering, or finishing, are usually required.

Metal matrix composites (MMCs), also known as ceramic reinforced metal matrix composites, are the mixture of metal matrix and ceramic reinforcements [22]. MMCs possess better thermo-mechanical properties and excellent chemical inertness as compared to traditional metals [2,25,26,27,28]. They are used under extreme operating conditions such as high load, elevated temperature, and extensive wear operations [2,25,29,30]. MMCs have gained wide popularity in the aerospace, biomedical, electronics, and various engineering applications [2,31,32,33,34,35]. They are difficult to process by conventional machining techniques due to high hardness and melting point [2,36,37,38]. Laser Additive Manufacturing (LAM) has opened, in the last few years, novel opportunities to develop MMCs for practical applications. Among the developed LAM techniques, SLS and LMD have been used to manufacture MMCs efficiently. Equipped with high energy density laser beams, LMD machines demonstrate their capability to process MMCs with high hardness and melting point. Another unique feature of the LMD is “cladding”, i.e., coating of a surface with a layer of metal or MMCs. This process is usually carried out on the surfaces of bulk (new/worn-out) materials with the emphasis on enhancing the surface characteristics or obtaining the desired biological, frictional, or chemical characteristics for a given material [2,31,32,33,34,35].

There are various established manufacturing methods, such as casting or milling for the production of MMCs. To their difference, LMD offers the possibility to synthesize the in-situ MMCs starting from powder precursors, to build a 3D object. Thus, some technological steps are eliminated, with a potential cost reduction of the fabricated part. Pressing or stirring during casting, therefore, become obsolete. Moreover, a big advantage for this synthesis route is the versatility when it comes to MMCs composition: the multilayer structures can be built efficiently using the same printing process. The high laser energy assists in attaining a very high temperature, resulting in dense parts. The synthesis of MMCs by LMD is not without defects. Since the metal is in a liquid state during laser irradiation, there is no control over the distribution of the particles in bulk after solidification. Moreover, effects such as cracks or pores can be widespread in LMD printing of parts.

To the best of our knowledge, this paper is the first review of literature related to 3D printing of MMCs by energy deposition techniques. We are gathering for the first-time data in literature related to additive manufacturing methods for these special materials, their mechanical properties after printing and which are the present and future trends. We organized the paper in three sections. In Section 1, different LAM processes have been described. The main emphasis has been laid on the LMD technique regarding working principle, and classification of the process parameters. The second section can be recognized as the core of the article. The following areas have been identified regarding MMCs: the difference between ex- and in-situ MMCs, various mixing techniques, powder- and wired-based LMDs, potentially identified matrices, properties of MMCs, multiple applications, potential challenges with their solutions, and future research direction. The third section provides the conclusion of this study.

## 2. Laser Additive Manufacturing (LAM) Processes

**Figure 1:**
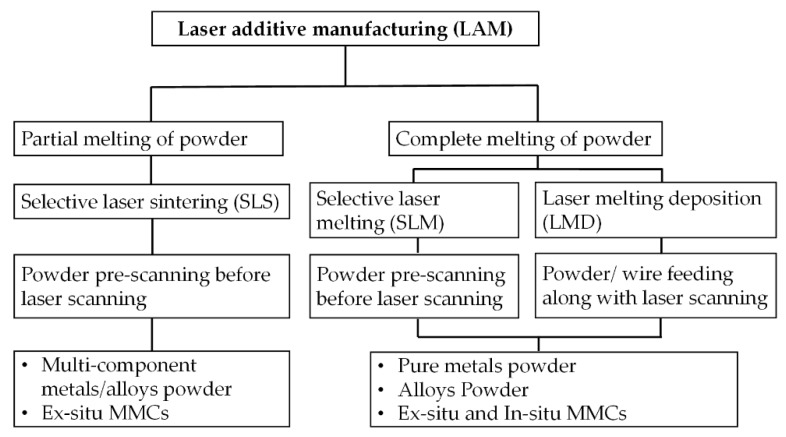
LAM processes classification based on laser-material interaction; based on the data in Ref. [22,39,40].

Figure 1 shows the classification of direct LAM techniques based on the laser-material interaction mechanisms. The LAM processes are classified into three basic methods: (a) selective laser sintering (SLS), (b) selective laser melting (SLM), and (c) LMD [22,39,40].

As this study is focused on the MMCs by LMD, therefore, an emphasis has been put on the LMD process.

### Laser-Melting Deposition (LMD) Process

**Figure 2:**
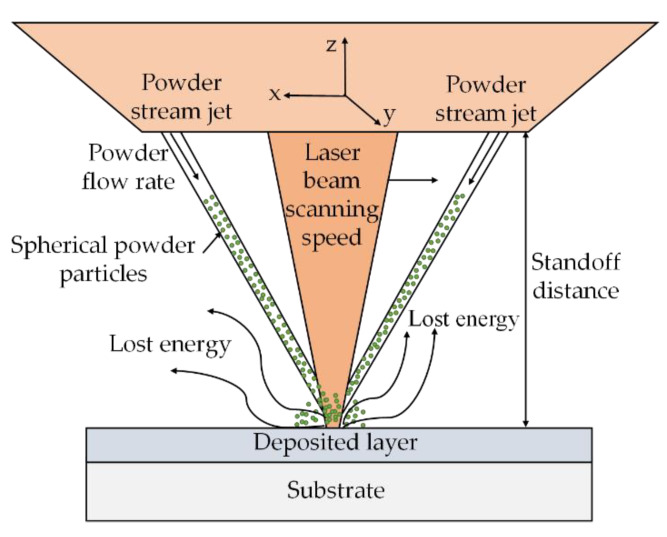
Schematic diagram of the LMD process; based on the data provided in Ref. [5,51].

Figure 2 presents the schematic explanation of the LMD process. Initially, the substrate is irradiated via a laser beam [41], generating a melt pool that captures and melts the powder particles blown by a nozzle. In LMD, various metal and ceramic powder particles can be mixed homogeneously to prepare different composites with better mechanical, structural and thermal properties [28,42,43,44,45,46,47,48,49,50,51,52,53,54,55,56]. The powder particles are carried and mixed by a jet of gases such as argon and helium [57]. As the laser source departs, the molten pool solidifies via heat dissipation by the conduction, convection, and radiation. The deposition head, containing the powder nozzle and optics for laser beam delivery, travels along the defined path, thus, forming a layer on the substrate. Afterwards, the deposition head moves upwards to the one-layer thickness for the deposition of the following layer. In the LMD, the first layer is partially re-melted, serving as a new “substrate”, thus, helping in the formation of the second layer. The same step is repeated until a 3D shape based on the CAD model is produced [5,51].

The LMD process can be classified into three techniques: (a) direct metal deposition (DMD), (b) laser-engineered net-shaping (LENS), and (c) direct light fabrication (DLF) [22]. It is important to note that the DMD, developed by Mazumder’s group [58], provides continuous closed-loop feedback to control the dimensional accuracy during the printing process. Therefore, a feedback setup is an exclusive characteristic that differentiates DMD technology from the rest of the technologies [58]. Table 2 introduces the classification of the LMD processes based on the deposition rate and technique, layer height, dimensional precision, and surface roughness.

**Figure 3:**
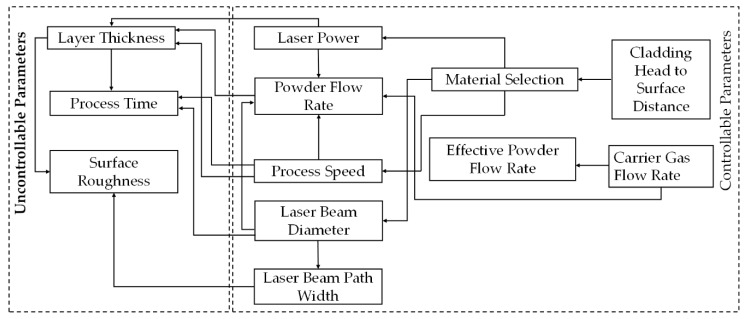
Process Parameters in the LMD technique; based on the data provided in Ref. [2,62,63,64,65,66,67,68,69].

The process parameters play a significant role in LMD process. Figure 3 classifies the process parameters: (a) controllable, and (b) uncontrollable parameters [62]. The controllable parameters can be tackled directly; however, the uncontrollable parameters can be adjusted indirectly through the controllable parameters [63,64,65,66,67,68,69].

**Figure 4:**
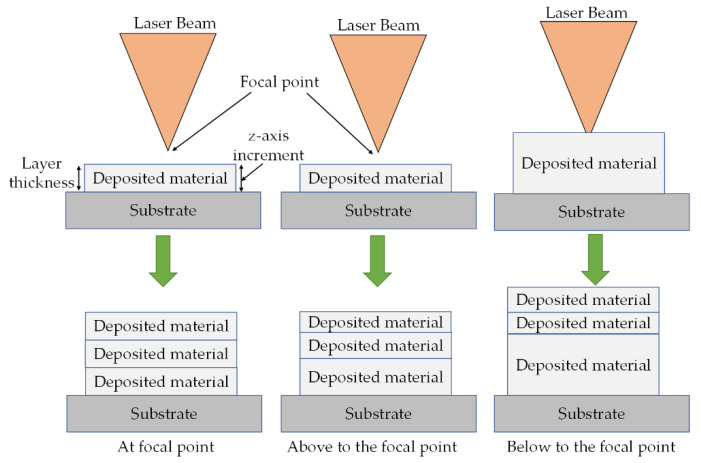
Correlation between growth along with the *z*-axis and layer thickness in LMD process; based on the data provided in Ref. [2,52].

A correlation between the growth along with the *z*-axis and layer thickness is essential as the misalliance between the deposited layer height and z-plane increment will cause extra-energy consumption and disturb dimensional accuracy, hence, resulting in poor quality part [2,52]. Figure 4 shows the relationships between growth, along with the *z*-axis and layer thickness. A good correlation between the *z*-axis increment and layer thickness will, therefore, result in uniform layer thickness, and the laser energy will be stable for all the layers, resulting in consistent properties of the whole fabricated part [2,52]. Hence, an optimum selection of operating parameters is necessary for proper use of energy density for dimensional control stability, as shown in Figure 4.

## 3. Metal Matrix Composites (MMCs)

**Figure 5:**
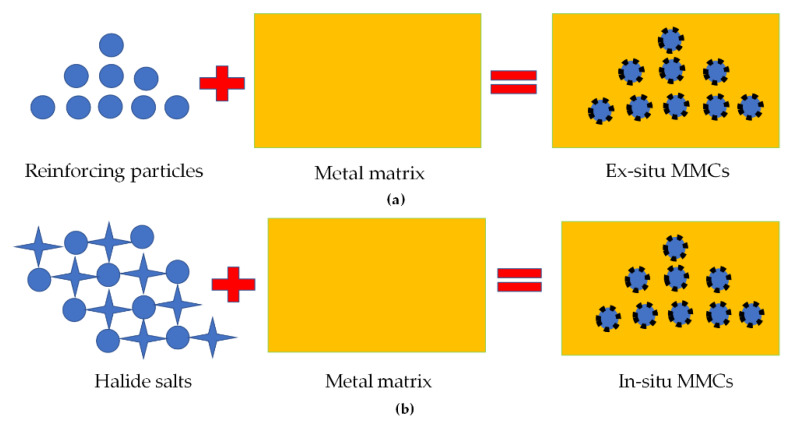
The graphical illustration of MMCs (**a**) ex-situ (**b**) in-situ; based on the data provided in Ref. [22,70].

MMCs are usually composed of a minimum of two types of materials, a metal matrix and a dispersed phase of metal, ceramic, or polymer [2]. They can be classified into ex- and in-situ MMCs [22,70]. Figure 5a presents the ex-situ MMCs process, in which the reinforcements, usually particulates, are manufactured and mixed externally into the metal matrix. In such MMCs, the reinforced in the form of particulates are repeatedly splintered and cold-welded, thus, making them potential candidates for the SLS process. On the other hand, Figure 5b exhibits that in-situ MMCs are manufactured by a chemical reaction between the halide salts and metal matrix, which are thermodynamically more stable than ex-situ MMCs. In-situ MMCs show compatible, strong interfacial bonding and better mechanical properties as compared to ex-situ MMCs. Such MMCs are the potential candidates for SLM and LMD processes. There are various advantages of the MMCs, but potential difficulties, including gas entrapment, particle accruement, and micro- and macro-cracks, play obstacles to produce fully dense MMCs.

### 3.1. MMCs Mixing Techniques

In the literature, various MMCs mixing techniques are available. The central perspective of these processing techniques is to achieve a standardized dispersal of the reinforcements within a matrix to achieve a defect-free microstructure. Table 3 collects the various MMCs mixing techniques.

In the case of LMD, the matrix and reinforcements, both are added in the powder form. Therefore, in this literature, more focus has been put on the powder metallurgy. Lanfant et al. [89] developed MMCs using Ti (matrix) + Al_2_O_3_ (nano-reinforcements) via the LMD process. A combination of Impakt powder and conventional powder feeding system was used to directly inject the Al_2_O_3_ nanoparticles into Ti-matrix to increase the hardness, locally. Mechanical tests were carried out; the results showed that the addition of Al_2_O_3_-nanoparticles (0–14 wt.%) within Ti-matrix could exponentially increase the hardness from 100 HV to 650 HV. Liu et al. [90] considered the tensile properties of TA15 titanium (matrix) + TiC (10 vol.% reinforcement) at high temperatures. These MMCs were pre-mixed in the form of powder blends. It was found that the MMCs exhibited higher tensile strength (UTS) and lower elongation as compared to the monolithic matrix alloys at 873 K. The UTS decreased from 625 to 342 MPa as the temperature increased from 873 to 973 K. In contrast, the elongation of the MMCs increased from 7% to 18%.

**Figure 6:**
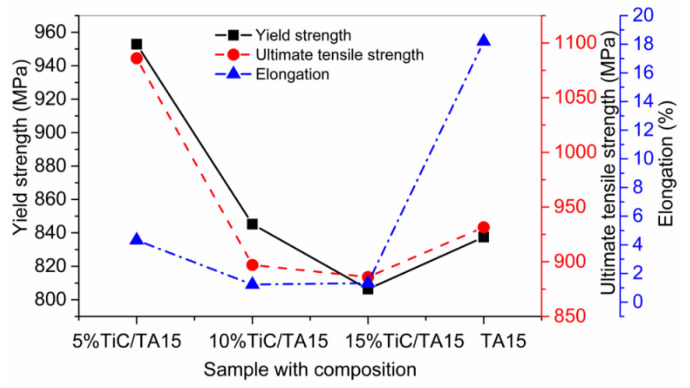
TiC + TA15 composites mechanical characteristics, including yield strength, ultimate tensile strength, and elongation; based on the data provided in Ref. [48].

Liu et al. [48] manufactured titanium matrix composites (TiC + TA15) by the LMD process. Initially, the TiC + TA15 in powder form was mixed with an acetone solution, and then the slurry was dried in an electric oven up to 393 K for two hours to eliminate the acetone solvent. The results are shown in Figure 6, which indicates that the specimen containing TiC (5 vol.%) presented better yield strength (YS) and UTS. In contrast, the tensile properties of the composites declined with the further increment of TiC (10 and 15 vol.%). The declination in the UTS of the composites is accredited to the premature failure of the TiC reinforcements. It can also be seen that pure TA15 presented better elongation (%) as compared to the composite ones.

Cooper et al. [91] manufactured MMCs with the addition of Al_2_O_3_ + SiC + TiC (5 wt.%) in the Inconel 625 matrix by the LMD process. A ball mill was used to mix the powders, at a speed of 150 rpm for one hour, until the powders appeared well-mixed visually. On the one hand, the material hardness increased up to 130%. On the other hand, the number of pores and cracks increased with the addition of SiC. In contrast, no appreciable effect upon material’s hardness was found with the addition of Al_2_O_3_.

### 3.2. Metal Matrix Composites (MMCs) Deposited by Wire and Powder Particles Feedstock

**Figure 7:**
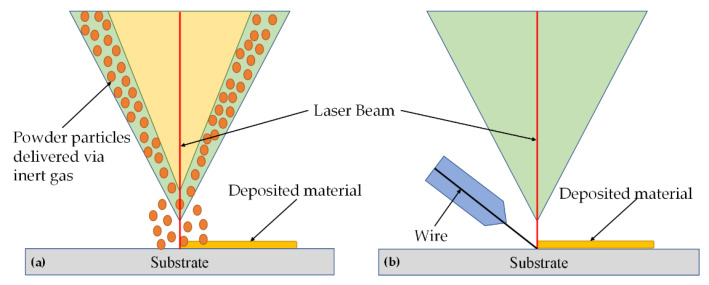
The LMD process: (**a**) coaxial powder feeding and (**b**) lateral wire feeding; based on the data provided in Ref. [92].

The LMD process can be further classified based upon the type of feedstock material. Figure 7 presents the two deposition techniques derived from the LMD process. In Figure 7a, the powder is fed coaxially along the laser beam while the lateral wire feeding into the melt pool can be observed in Figure 7b [92].

Processes, as mentioned earlier, own some pros and cons, which are summarized in Table 4 [93,94,95,96,97].

The pros and cons of combined powder- and wire-based LMDs are presented in Table 5 [92].

**Figure 8:**
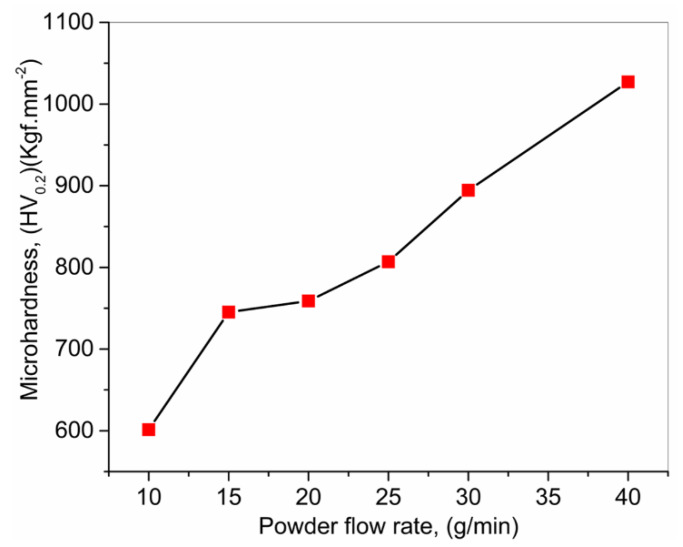
Effect of WC powder flow rate on microhardness; based on the data provided in Ref. [98].

Farayibi et al. [98] investigated the LMD of Ti6Al4V (wire) + tungsten carbide (WC), in powder form, fed concurrently into the melt pool generated on a Ti6Al4V substrate via the laser beam. The WC particulates participated as the strengthening agent within Ti6Al4V, thus, improving the deposited MMCs’ hardness and wear resistance. Figure 8 shows the microhardness of the deposited material, in which the powder flow was increased (10–40 g/m), while the wire feed rate kept constant (800 mm/min). It can be analyzed that with the increment in powder flow, the hardness of the deposited MMCs rises significantly (600–1000 HV).

Farayibi et al. [99] developed a new MMC using Ti6Al4V + titanium diboride in the powder form via a satelliting method. The resulted mixture consisted of large Ti6Al4V particles, enclosed within finer needle-shaped TiB structures, presented an improved hardness. The microstructural characterization exposed that the composite consisted of TiB eutectic precipitates in an α+β-Ti-matrix. They contained the incompletely melted Ti6Al4V + TiB_2_ particulates. The satelliting of TiB_2_ particles onto Ti6Al4V surfaces notably enhanced the dispersion of the composite, which was characterized by the arbitrarily oriented and uniformly distributed TiB needles within the microstructure. The composites’ hardness, when fabricated by this technique, was in between 440 to 480 HV.

### 3.3. Different Laser Sources for In-Situ MMCs Syntheses by LMD

Various studies to manufacture MMCs by a unique laser source have been proposed. Ramakrishnana and Dinda [100] manufactured MMCs using Al (82 wt.%) + W (18 wt.%) via the LMD technique. A laser beam (diode laser Laserline LDM 2000-40, 978 nm) of 900 W, in combination with three scanning speeds (1.5, 6, and 12 mm/s), was used for the MMCs fabrication. The hardness of the developed MMCs increased up to 50% in comparison with pure aluminum. A finer microstructure was found at 12 mm/s scanning speed as compared to 1.5 and 6 mm/s scanning speeds. Zhenglong et al. [101] prepared TiB_2_ (particulate) + AA7075 (matrix) MMCs using the LMD technique. A laser beam (IPG fiber laser, YLS-5000) with 800 W and 10 mm/m scanning speed was used in the process. The results exhibited that the grain dimensions decreased after adding TiB_2_ due to heterogeneous nucleation. In comparison to the unreinforced AA7075 sample, TiB_2_ + AA7075 MMCs showed an elevated hardness. The hardness of the MMC with TiB_2_ (4 wt.%) was found to be 127.8 HV, while the grain size was reduced up to 16.8 μm.

Li et al. [55] designed and fabricated novel MMCs using α-Fe, vanadium (V), victorium (VC), and chromium carbides (Cr_7_C_3_, Cr_23_C_6_) via the LMD process. The V was used in 9, 12, and 15% (wt.%), respectively. For fabrication, a ytterbium laser source with 2.2 kW and 8 mm/s scanning speed was used. The results indicated that the VC particles provided adequate space for the heterogeneous nucleation of α-Fe and Cr_23_C_6_, during the solidification of the molten material, which resulted in refined grain structures. The microhardness of the three specimens was in the range of 521–603 HV. The sample having V with 12 (wt.%), resulted in the lowest wear rate (5.011 × 10^−6^ mm^3^/Nm) among all the tested samples.

### 3.4. Matrices for MMCS

#### 3.4.1. Titanium-Based MMCs (TMCs)

Titanium alloys have various aerospace, marine, biomedical, structural and industrial applications. They owe excellent strength to weight ratio, malleability, formability, deterioration resistance, and biological compatibility characteristics. However, these alloys yield reduced hardness and wear attributes. A suitable way to increase the hardness and tribological characteristics of Ti-alloys is to mix the Ti-matrix with tough precipitates to achieve Ti-based composites (TMCs). These can be classified into two sub-categories based on the type of reinforcement: (i) continuous reinforced TMCs (ii) discontinuous (particles) reinforced TMCs [102,103,104]. Table 6 summarizes the continuous and discontinuous TMCs formation techniques regarding illustration, advantages, and disadvantages.

In the last few years, the discontinuously strengthened Ti-composites have experienced rapid expansion. They have an elevated strength, toughness, wear resistance, and thermal reliability as compared to pure Ti-alloys. Therefore, they are potentially considered for applications, including aerospace and automotive. Different particulates have been selected for reinforcement so far such as TiB_2_, TiN, B_4_C, ZrC [42,104], nano-SiC [114], TiB, TiC [115,116], Al_2_O_3_ [117], and Si_3_N_4_, which were found unstable due to the formation of titanium silicide and carbon nanotubes [118,119,120]. For the continuous fibers, TMCs using boron reinforcing fibers (coated with silicon carbide) were produced; however, these fibers are expensive as compared to the other fibers, which leads to discontinuation of TiB fibers [104,121,122]. Besides this, various researches have been carried out using SiC [123,124,125,126,127], carbon [104,128,129], SCS-6 and Sigma [130,131] reinforced fibers.

**Figure 9:**
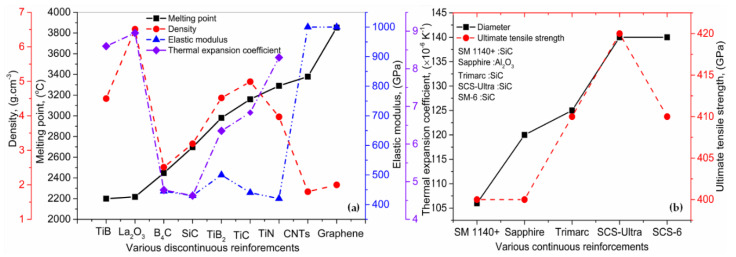
Properties of various TMCs (**a**) discontinuous and (**b**) continuous reinforcements; based on the data provided in Ref. [104].

Figure 9a displays the mechanical and physical properties of various discontinuous TMCs. It can be seen that the TiB and graphene present the least and highest melting point, respectively, as compared to the other discontinuous reinforcements (DRFs). It means that a low amount of laser energy will be needed to melt down the TiB. In contrast, an opposite behavior can be observed for graphene. Moreover, La_2_O_3_ presents the highest density, while graphene possesses the least density value. To the best of our knowledge, the elastic modulus of TiB and La_2_O_3_ containing MMCs are not reported in the literature, which identifies the potential area for future research. The B_4_C, SiC, TiB_2_, TiC, and TiN have almost the same elastic moduli; but, the carbon nanotubes (CNTs) and graphene present the highest elastic moduli as compared to the rest of DRFs. Similarly, the La_2_O_3_ has the highest thermal expansion coefficient as compared to the rest of DRFs. Figure 9b shows a comparison of various continuous reinforcements (CRFs) regarding their diameter and ultimate tensile strength (UTS). The two major types of CRFs, including SiC and Al_2_O_3_, have been presented. The majority of the CRFs such as SM 1140+, trimarc, SCS-ultra, and SM-6 belong to the SiC category, while sapphire belongs to the Al_2_O_3_ type. The output explains that SCS-ultra, with a diameter of 140 µm, shows the maximum UTS. In contrast, for the rest of the CRFs, a compromise should be reached between the particulate size and UTS. By keeping in view the trend presented in the Figure 9, one can conclude that the proper selection of DRFs and CRFs has to be made based on the specific requirements, and a compromise should be made between the thermal and physical properties [104].

#### 3.4.2. Nickel-Based MMCs (NMCs)

Ceramic reinforced nickel matrix composites, also known as NMCs, usually possess high fatigue and corrosion resistance with better hardness and wear resistance properties as compared to the simple nickel matrix. They are considered to be prospective materials in aerospace, biological, and petrochemical manufacturing due to above-mentioned properties. TiC strengthened NMCs via LMD were produced by Hong et al. [132]. They found that the existence of TiC was beneficial to refine grain size, from 34.1 µm to 27.2 µm. Moreover, the results displayed that the high energy can lead to an effective Marangoni convection confined by the melt pool, which induces refined and normalized spreading of TiC reinforcements. Thus, resulting in improved wear resistance and malleability. Furthermore, an increment in the thermal energy input per unit length resulted in uneven columnar dendrites formation, declining the wear and tensile properties of NMCs. Li et al. [133] produced Ni + TiC NMCs by LMD. A total of three TiC compositions, including 20, 40, and 60 (vol.%), were selected. The influence of TiC vol.% on phase transformation, microstructure evolution, hardness, and wear resistance, was analyzed. The analyses exhibited that the composites consisted of TiC + Ni phases, demonstrating that TiC was produced through in-situ reaction. Moreover, TiC particulate size increased from 3 to 10 μm, when the TiC (vol.%) was enhanced from 20 to 60%. In addition, the hardness was improved from 365.6 HV_0.3_ to 1897.6 HV_0.3_, while the value of wear resistance changed from 20 to 6 × 10^−3^ g.

#### 3.4.3. Other Metal Matrix Composites

In the LMD process, the rapid heating and solidification lead to the warpage, delamination, and cracks. Li et al. [134] carried out a study on the usage of invar. The experimental results showed that TiC reinforced invar, with the 64 wt.% Fe and 36 wt.% Ni composition, has a low thermal expansion coefficient, improved rigidity, and YS. Xiong et al. and Picas et al. [135,136,137] manufactured WC + Co using LENS. An improved microstructure, wear resistance, and mechanical properties were found with the addition of WC in Co. Choi and Maumder [138] reported a study on the manufacturing of Fe + Cr + C + W MMCs via the DMD, thereby, producing an innovative wear-resistant material. The results explained that the conformation and volume proportion of carbides could easily be handled by regulating the pre-heating temperature, input power density, and scanning speed. The matrix participation was carried out by M_6_C and M_23_C_6_ type carbides; the rhombus-shaped M_6_C carbides showed better tribological properties. Zhong et al. [139] synthesized the Ni-Al intermetallic layers and TiC (particulates) MMCs by laser cladding. The powder particles were added coaxially. The printed layers were cracks free and metallurgically bond to the substrate. The microstructure of the layers was mainly composed of β-Ni-Al phase and a few γ-phases. Moreover, un-melted dispersive fine precipitates of TiC particles and refined β-Ni-Al phase matrix were found in the composites. The hardness test shows that the microhardness for Ni-Al intermetallic layers was equal to 355 HV_0.1_, and 538 HV_0.1_ for Ni-Al+ TiC matrix composites.

### 3.5. Properties of MMCs

MMCs present remarkable characteristics, which makes them applicable in the fields of aerospace, automotive, heavy goods vehicles, electrical, and biomedical [140]. Few properties of MMCs are reviewed below.

#### 3.5.1. Mechanical Properties: Hardness, Ultimate Tensile Strength (UTS), Yield Strength (YS), Elongation, and Wear

**Figure 10:**
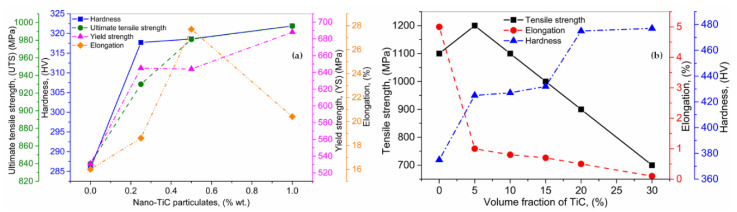
(**a**) Influence of Nano-TiC particulates on the hardness, ultimate tensile strength, yield strength, and elongation, and (**b**) the consequence of TiC (vol.%) on Tensile strength, elongation, and hardness; based on the data provided in Ref. [56,142].

MMCs present superior properties including hardness, YS, and UTS in comparison to the base alloys. Li et al. [141] manufactured MMCs through the LMD by feeding the WC powder particulates along with titanium wire into the melt pool generated by the laser beam. Major process parameters, including wire feeding rate, powder flow, and the laser power were used in the analyses. The microhardness of the MMCs was 500 HV_0.2_, which is much higher than the titanium alloys (320 HV_0.2_). It indicates that the presence of the WC + TiC phases within the printed layer improved the hardness and abrasive resistance. Bi et al. [142] carried out the deposition of inconel 625 + TiC nano-powders using the LMD. The mechanical properties were investigated. Three different compositions of TiC + inconel 625, including 0.25/99.75, 0.50/99.50 and 1.00/99.00 (wt.%), were synthesized. The hardness, UTS, YS, and elongation are shown in Figure 10a. It can be seen that the hardness, UTS, and YS increased proportionally with the increment in the quantity of TiC particulates (wt.%). However, elongation presented random behavior with the increment in TiC particulates (wt.%). The maximum elongation was exhibited by 0.50/99.50 composition. Gopagoni et al. [143] processed Ni (80 wt.%) + Ti (10 wt.%) + C (10 wt.%) MMCs via LENS technique. The manufactured MMCs showed the eutectic TiC and FCC-TiC structures. The tribological and mechanical analyses were conducted. The stationary friction coefficient was found equal to 0.50, inferior to pure Ni. Moreover, the hardness increased substantially up to 370 VHN, proving them a potential candidate for surface engineering operations. Crack-free functionally graded MMCs composed of TiC particulates + Ti6Al4V were manufactured by Wang et al. [56] using LMD. A volume fraction of TiC in between 0–30 (% wt.) was used to analyze the effect of TiC (vol.%) on the microstructure and mechanical characteristics of MMCs. They found that the hardness gradually increased with the increment in TiC (vol.%), which can be attributed to the presence of eutectic + TiC phases. When TiC increased up to 5 (vol.%), the tensile strength enhanced by 12.3% as compared to the Ti6Al4V alloy. Nevertheless, the tensile strength and elongation of the produced MMCs decline as the volume fraction of TiC surpass by 5%. It can be explained that the number of stiff un-melted TiC particulates, amount and dimension of dendritic TiC phases raised with the increment in TiC. These results are presented in Figure 10b.

**Figure 11:**
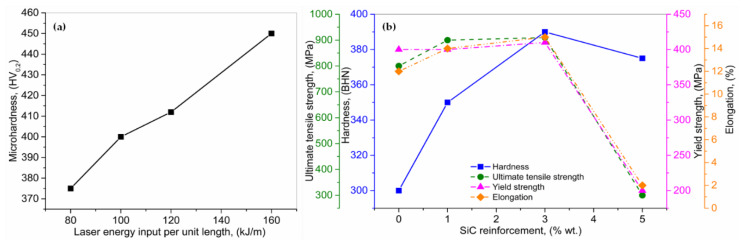
(**a**) Analyses of laser energy input over the unit length on microhardness, and (**b**) influence of SiC reinforcement (wt.%) on hardness, ultimate tensile and yield strengths, and elongation; based on the data provided in Ref. [144,145].

Hong et al. [144] used the LMD to manufacture inconel 718 + TiC MMCs. The influence of the laser energy over the unit length (80–160 kJ/m) on the microstructures and hardness, was analyzed. The TiC experienced a tremendous transformation as the laser energy density increased from 80 to 120 kJ/m. The comparatively coarse polyhedral TiC particulates resulted when the laser energy was up to 100 kJ/m. As the laser energy increased beyond 100 kJ/m, completely liquified smooth TiC particulates were produced. Furthermore, when the laser energy increased beyond 160 kJ/m, the TiC particulates were significantly refined. However, a direct relationship was observed between laser energy input and microhardness for the produced MMCs, as given in Figure 11a. Sateesh et al. [145] manufactured MMCs using pre-heated nickel phosphide coated with SiC reinforced particles via the LMD process, under inert nitrogen atmosphere. An inclination in the hardness, UTS, YS, and elongation was observed with the increment in SiC (wt.%). These properties decline dramatically beyond 3 (wt.%) addition of SiC. For a given laser scanning speed and power, the increment in SiC (wt.%) resulted in excessive un-melted SiC particles, leading to the declination of hardness, UTS, YS, and elongation, as shown in Figure 11b.

**Figure 12:**
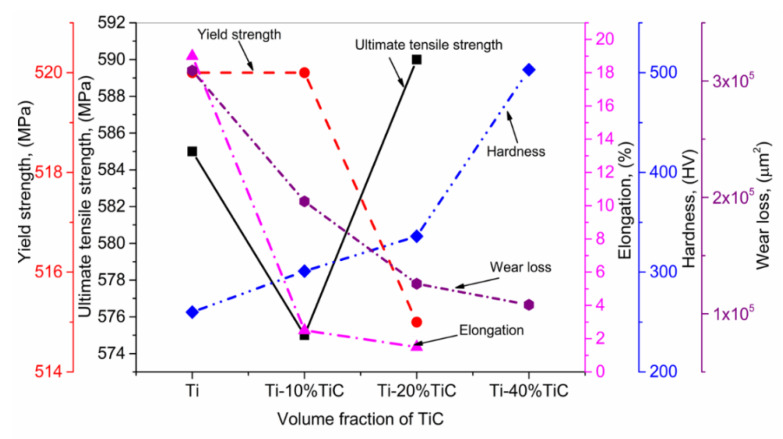
Effect of TiC reinforcement (vol.%) on ultimate tensile and yield strengths, elongation, hardness, and wear loss; based on the data provided in Ref. [146].

Zhang et al. [146] used the LMD to manufacture Ti + TiC MMCs with various pre-mixed ratios of TiC (10, 20 and 40 vol.%) The mechanical testing, including UTS, YS, elongation, hardness, and wear, were conducted. The results are presented in Figure 12. It can be observed that the UTS slightly deviates with the accumulation of TiC; meanwhile, YS declines quickly, which is caused by the existence of solid and stiff TiC particulates. The hardness and wear resistance of the produced MMCs raised with the increment in TiC volume fraction due to the strengthening effect of the particulates and the optimum bonding between TiC and Ti. Based on the excellent adhesion between TiC + Ti MMCs, such coatings can be deposited on the surfaces, where superior wear resistance is required. The LMD process inherits the high laser energy density; moreover, Ti showed an excellent affinity to the Oxygen + Nitrogen, which was absorbed quickly in the interstitials. The combination of the aforementioned facts leads to strength increment. Furthermore, a declination in the elasticity was observed in the MMCs.

Table 7 provides a summary of the above-mentioned mechanical properties.

#### 3.5.2. Creep Behavior, Erosion Resistance and Thermophysical

**Figure 13:**
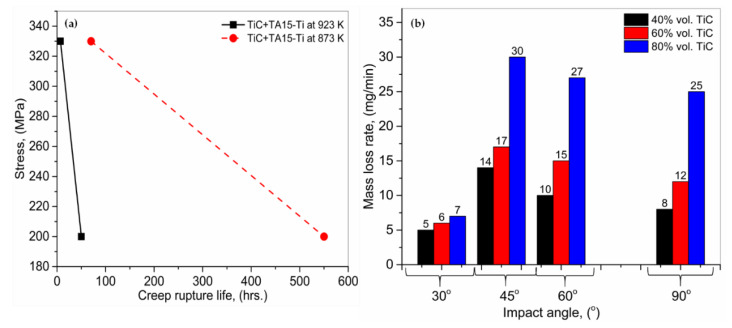
(**a**) Creep behavior of TiC + TA15-Ti MMCs, and (**b**) erosion rates for 40, 60, and 80 (vol.%) TiC + H13 tool steel MMCs at various impact angles; based on the data provided in Ref. [147,148].

Liu et al. [147] analyzed the creep performance of TA15-Ti + TiC particulates (10.8 vol.%) MMCs at 873 and 923 K, respectively, deposited by the LMD. The creep resistance of the MMCs improved notably due to the accumulation of TiC reinforcing particulates in comparison to the monolithic TA15-Ti alloy designed in between 723 to 773 K. The creep life for TA15-Ti + TiC MMCs at 873 and 923 K, is shown in Figure 13a. The rupture of the TiC + TA15 MMCs was originated due to the particles’ cracking, interfacial debonding and voiding, which becomes dominant with the temperature elevation. Jiang and Kovacevic [148] performed a study on the behavior of TiC + H13 tool steel MMCs, manufactured by LMD. The influence of TiC (vol.%) on erosion resistance was analyzed. Figure 13b shows the erosion rates of the coated layers at different impact angles. For all the coatings, it can be observed that the impact angle influenced the erosion rate considerably. For the 30° impact angle, the lowest erosion rate was observed, followed by a 90° impact angle. The maximum erosion was found at 45° and 60° angles. Moreover, TiC with 80 (vol.%) presented the least erosion resistance, while the coating with 40 (vol.%) showed the highest ones.

**Figure 14:**
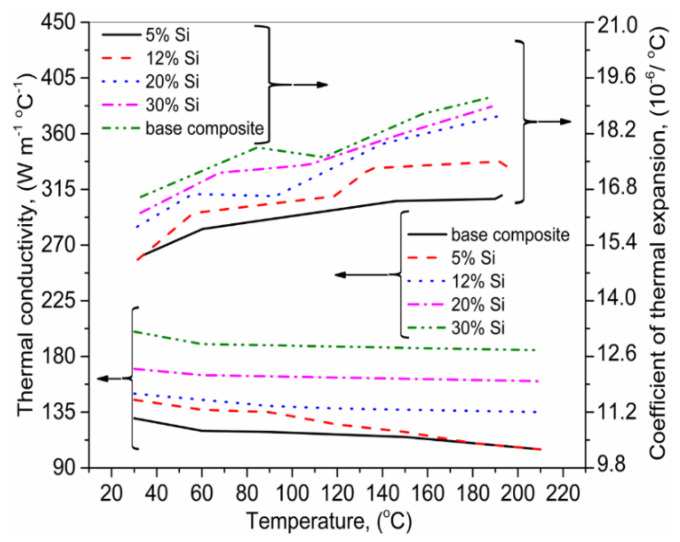
The relation between temperature and various fractions of Si on thermal conductivity and thermal expansion coefficient; based on the data provided in Ref. [149].

Lei et al. [149] used the LMD for Si_p_ + 6063Al MMCs for 5, 12, 20 and 30 (wt.%) Si contents. The thermophysical properties of MMCs were investigated. The results are shown in Figure 14. They found that with the increment in Si (wt.%), the thermal conductivity of the MMCs declined due to the decrease in an α-Al phase, which exhibits the high-level conductivity. The thermal expansion coefficient presented an opposite behavior as compared to the thermal conductivity.

### 3.6. Applications of MMCs

The literature survey revealed the following main areas proposed by various studies.

#### 3.6.1. Biomedical

MMCs have outstanding load-bearing and wear resistance properties, which make them interesting for implant applications [150]. TiN and SiC reinforced TMCs [151,152] have been proved for excellent biocompatibility. Hence, they are used for cladding on the metallic substrates to upgrade the biocompatibility of implants. In vitro testing was carried out to assess the biocompatibility of the deposited layers. The results showed an excellent cell to material interaction, and no toxicity was found. It showed that they could be used for load-bearing implants such as hip, knee and shoulder joints [151,152]. Moreover, orthopedic bone prototypes and meshed cranial prostheses implants were successfully developed for biomedical applications [57,153,154].

#### 3.6.2. Wear Resistance

In MMCs, the ceramic reinforcements within the matrix play as the load-bearing element, which can confine the plastic distortion, thus, preventing the matrix from deterioration. It makes MMCs a potential candidate for wear-resistant applications [5,155]. The depositions of TiN reinforced MMCs on a Ni-Ti substrate by LMD, in a nitrogen atmosphere, increased the substrate’ wear resistance by a factor of two [156]. TiB + TiN MMCs were prepared on a Ti-substrate using pre-mixed boron nitride (BN) + Ti6Al4V powders by the LMD. With the increase in the BN content up to 15 (wt.%), the surface hardness increased from 543 to 877 HV, thereby, increasing the wear resistivity of the deposited layers [157]. The deposition of WC/W_2_C + Ni MMCs on steel substrate was carried out via LMD. It was found that an inclination in the WC/W_2_C content and a declination in carbide dimension, can enhance the wear resistance of MMCs up to 200 times more than the pure Ni matrix [158]. The dry friction, along with better contact load, is an increasing demand for aerospace and heavy industries. In simple words, only the high wear resistance is not sufficient. For this difficulty, WC/Co (ceramic reinforcement) + Cu-Sn (solid lubricant) were manufactured by the LMD process. The developed structures presented better wear resistance and low frictional characteristics [159].

#### 3.6.3. Corrosion and Erosion Resistance

MMCs are used to increase electrochemical corrosion and erosion resistance. For this purpose, Ni_2_Si composites were deposited on a steel substrate by the LMD technique [160]. Immersion and anodic polarization tests were conducted, which proved the deposited layer intermediated better bio- and electrochemical corrosion resistance. The novel layers of TiC + Satellite 6, WC + Co, MoSi_2_ + stellate 6, and MoSi_2_ + steel matrix composites, were deposited on a metallic substrate to improve erosion wear rate [161].

#### 3.6.4. Industrial

LMD technology has recently been used to repair brake disks and turbo-engine parts [162]. In one of the recent studies, LMD was demonstrated as a potential candidate to repair steel dies using the Fe-Cr and Fe-Ni layers [163]. Moreover, various applications of the LMD have been determined in the fields of aeronautics and refractory [164]. Furthermore, LMD technique has been tested for the manufacturing of multiple parts, including turbine and compressor blades [165], nozzle guide vanes [166], jet engines [167], casting dies [168], Z-notches [169], bearing seats, valves, shafts, cylinders and rods [170], and seals [171].

### 3.7. MMCs by the LMD: Strengths, Challenges and Their Potential Solutions

On the one hand, MMCs produced by LMD show excellent hardness, toughness and frictional properties. On the other hand, MMCs loose ductility, YS, and UTS with the addition of ceramic particulates. Mechanical properties of MMCs primarily depend on the adhesion between ceramic reinforcements and metal matrices along with the interface. Zheng et al. [172,173] used an effective strategy to overcome such difficulties, by encapsulating ceramic particles within metallic coatings to strengthen the inconel-625 + Ti-6Al-4V MMCs. This method prevents the bunching of ceramic particles within MMCs, thus, effectively reducing the voids and cracks formation in between metal and ceramic intersection. There are various hindrances for obtaining the fully dense and homogenized MMCs. The dense MMCs are restricted due to micro/macro-cracks, gas entrapment, and particulate accumulation during the printing process.

Moreover, if the initially deposited layers are poorly bonded to the substrate, the following layers deposition will result in an up-warp, hence, resulting in manufacturing failure [174]. Due to rapid heating and solidification in the LMD process, cracks are usually induced due to the large thermal gradient [175]. These cracks decrease the lifetime of the fabricated parts. The cracks can cause the catastrophic failure of the deposited layers under cyclic loading [103]. Therefore, it is extremely important to manufacture fully dense parts [28,176,177]. Various methods can solve the problems as mentioned above: (a) Process optimization [178,179], (b) integrating the LMD process with assisting technology such as ultrasonic vibration [180,181], (c) pre/post-heating the substrate to decrease thermal gradient [182,183], (d) adding rare earth oxides to change the melt pool dynamics [184,185] and (e) tailoring the novel structures [186,187].

### 3.8. Future Research Directions in MMCs

Based on the current review, a few potential areas still need attention from the researchers. In the LMD process, strong bonding between the deposited MMCs layers with the substrate is of great importance to fabricate bulk parts. One of the ways to achieve the desired strength is “process optimization.” However, in situations where process optimization fails, integrating an assisting technology such as ultrasonic vibration with LMD can be a decent alternative to secure an optimum bonding between the deposited layers and substrate.

Moreover, the post-processing needs special tools and high energy, thus, increasing the fabrication cost. This high cost limits the availability of MMCs to niche applications. There is a need to find out a balanced solution between better thermo-mechanical properties and low production cost, which can promote the MMCs effectively.

Depending on the amount of the dispersed phase within the composition, MMCs can display new properties or an enhancement of the existing ones. By simultaneous addition of matrix and reinforcement in powder form, might result in new materials with exciting properties. In addition, parts with complex architecture such as multilayered structures or gradient composition can be easily obtained via in-situ MMCs. Another area to explore is to manufacture the exact composition of MMCs via different laser sources as it may affect microstructure, mechanical, thermal and electrical properties significantly.

LMD is not suitable for the parts with fine geometry in the range of hundred-microns. On the other hand, SLM is a technique appropriate for metal parts with lattice structures and complex geometries. However, LMD scanning heads future developments can allow the obtaining of sub-mm resolutions. Hence, an increase in resolution for the MMCs printing via the LMD is a new area to be explored.

The newest trend in 3D printing is the use of enhanced topology. The CAD/CAM user specifies the part size, restrictions and the acting forces into the dedicated software. With this data, the software calculates and proposes the best shape regarding user’s requirements and the maximum resistance to the forces that will act upon it. However, the shape offered by the software is most of the time, unconventional and convoluted. Conventional casting or pressing techniques are often inappropriate for producing it, while 3D printing, in this case, is a suitable choice for building such parts. LMD printing method can advance this field even more by involving MMCs.

## 4. Conclusions

LMD process, depending on the experimental setup can be classified into three sub-categories: (a) DMD, (b) LENS, and (c) DLF. Every process has its own pros and cons and is selected depending on requirements of specific applications.

In the LMD technique, process parameters can be classified in two main categories: (a) controllable and (b) uncontrollable. The controllable parameters, such as laser power, scanning speed and powder flow rate, can be tackled directly. In contrast, the uncontrollable parameters, including layer thickness, process time and surface roughness, can be indirectly adjusted through the controllable parameters. Furthermore, a good correlation between the *z*-axis increment and layer thickness is necessary for uniform layer thickness and the optimal energy utilization. The operating parameters define the melting degree and properties of the fabricated parts. In addition, the side effects caused by high thermal stresses, resulting in part distortion can be lessened by process optimization. Hence, a proper selection of operating parameters is necessary to achieve high-quality parts.

MMCs are usually composed of a minimum of two types of materials, a metal matrix and a dispersed phase of ceramic, or polymer. The most widely used materials for matrices are titanium, nickel, invar, cobalt, and aluminum. Extensive research has been conducted on titanium matrix as it is a bioinert material that can be used in implantology. Invar, cobalt, and aluminum provide the potential research area to be further explored. Common reinforcements are hard ceramics such as carbides, nitrides, borides, oxides and graphene, mostly in form of microparticles.

MMCs deposited by LMD present extraordinary features such as high strength at elevated temperature, improved hardness, better fatigue, and creep characteristics in comparison to the traditional alloys. It makes them potential candidates for advanced technological applications that require in situ manufacturing of parts. In contrast, the opposite behavior for elongation percentage has been observed. UTS and YS show random behaviors. It can be explained that the interfacial bonding between the matrix and reinforcement is mandatory to achieve better UTS and YS. A weak adhesion, between the two phases restricts the load transfer from the matrix to reinforcement, thus, causing a declination in UTS and YS. Moreover, a wise selection of reinforcement and their fraction (wt.%) in combination with the metal matrix is necessary to achieve optimum physical, thermal, and mechanical properties.

During the fabrication of MMCs by LMD, cracks are usually caused by the enormous thermal gradient. These cracks decline the mechanical properties and shorten the lifetime of the fabricated parts. Process optimization, pre-heating, or introducing an assisting technology, has reduced the cracking problems.

LMD printing using MMCs still has a lot of open themes for future research. The problem of interface between the matrix and the dispersed phase is not yet solved. There is the emergence of new software that will revolutionize 3D printing by allowing the print of parts with enhanced topology. The research is underway for the complex architecture of parts using multilayers and gradient compositions of MMCs; there is still a potential for the discovery of new materials and properties.

The authors express their confidence that by joining two cutting edge research fields such as 3D printing and synthesis of prospective superior materials represented by MMCs will ensure not only years of research and possible breakthroughs for both scientific communities but also the emergence of new products with benefits for humanity: lighter vehicles, longer-lasting mechanical tools, more resistant implants and prostheses, better sports equipment, new construction materials and components for aeronautics and space programs.

## Figures and Tables

**Table 1:** Classification of AM Processes.

AM Processes	Processes	Layer Forming Principle	Forming Material	MMCs Application	References
Direct-AM Process	Selective laser sintering (SLS)	Partially melting by laser	Powder	Yes	[6,7]
	2. Selective laser melting (SLM)	Complete melting by laser	Powder	Yes	[8,9]
	3. Laser-melting deposition (LMD)	Complete melting by laser	Powder/Wire	Yes	[10,11]
Indirect-AM Process	Fused deposition modelling (FDM)	Extrusion	Filament	Yes	[12,13]
	2. Stereolithography (SLA)	Photo curing via laser scanning	Resin and powder	No	[14,15]
	3. Direct inkjet printing (DIP)	Inkjet printing	Powder suspension	No	[16,17]
	4. Layer-wise slurry deposition (LSD)	Slurry deposition	Slurry	Yes	[18,19]
	5. Laminated object manufacturing (LOM)	Sheet binding and laser cutting	Sheet	Yes	[20,21]

**Table 2:** Types of the LMD process.

Method	Feedback Loop	Deposition Technique	Layer Height (µm)	Deposition Rate (cm^3^/min)	Dimensional Precision (mm)	Surface Roughness (µm)	References
DMD	Available	Cladding via laser beam	250–254	0.99–4.00	N/A	38–40	[59]
LENS	Not available		129–381	N/A	XY-aixs = ±5, *Z*-axis = ± 0.40	59–93	[60]
DLF	Not available		195–200	1.0	±0.13	18–20	[61]

**Table 3:** Mixing techniques for MMCs.

Technique Name and Description		Essential Features	References
Stir Casting This technique involves the integration of ceramic particulate (reinforcements) into a liquid metal matrix by stirring mechanically and allowing the mixture to solidify.		(a) The dispersion of reinforcements is limited up to composites’ 30 wt.%. (b) The reinforcement will not be homogeneous in the matrix if it is more than 30 wt.%. (c) The clustering of reinforcement is difficult to avoid. (d) Wettability is challenging to maintain. (e) Segregation of reinforcing particles during floating takes place due to the density difference between the matrix and reinforcements. (f) Low-cost process.	[71,72,73,74,75]
Rheocasting In this process, the reinforcing particulates are mixed into the matrix, usually a metal. The given matrix is in between the solidus and liquidus temperature. The reinforcing particles are entrapped within the matrix, mechanically.		(a) An adequate bonding is achieved between the reinforcing particles and liquid matrix. (b) It results in better distribution of reinforcing particles, less porosity, improved wettability, and lower volume shrinkage. (c) It is a most reasonable method for fabricating the composites with discontinuous fibers or particulates. (d) It yields better distribution and integration of the reinforcing particles within the matrix as compared to the stir casting process.	[76,77]
Squeeze casting In this technique, the pressure is applied and maintained until the molten metal solidifies. The applied pressure assists in grain refinement that ultimately enhances the mechanical properties of the final product.		(a) In this technique, the rapid solidification of the part is achieved. (b) Excellent strength and ductility are obtained due to the rapid solidification process. (c) Gas porosity and shrinkage cavities are drastically reduced, resulting in excellent properties.	[78,79,80,81]
Powder metallurgy In this technique, a blending of fine powder particles, compacting into an anticipated form. Mostly, material heating is also involved.		(a) It is usually used for matrices with a high melting point. (b) It avoids segregation and brittle product formation. (c) It can produce complex shapes with satisfactory dimensional accuracy. (d) It yields minimum material loss. (e) Less secondary machining operations are needed. (f) The manufactured parts are relatively defect free. (g) It can incorporate a high-volume fraction of reinforcement.	[82,83]
Advanced shear technology This process uses the melting-condition advanced shear technology technique. A sufficient quantity of shear stresses is applied to the particles, within the liquidus metal, to get over the cohesive force and the malleable strength of the given mixture. It consists of the following mixing steps:		(a) Near-net shaped MMCs are produced with homogeneously distributed reinforcements. (b) It can yield MMCs with suitable microstructures. (c) The standardized dispersal of the reinforcement within the matrix is achieved. (d) Excellent mechanical properties are usually achieved, as compared to other techniques.	[84,85]
*Step I: Distributive mixing*	*Step II: Dispersive mixing*		
It employs the conventional mechanical stirring to pre-mix the metal matrix with the reinforcing particles. The equipment is the same as stir casting.	In this step, adequate shear stress is applied to overcome the average tensile strength of the agglomerated structures.		
Ultrasonic assisted casting It is a well-known process to produce lightweight nano-metal matrix composites (NMMCs) with excellent reinforcement distribution. However, NMMCs present severe problems regarding the uniform dispersion in liquid metal that induces clustering. This drawback can be solved by integrating the ultrasonic system with the casting process.		(a) It can produce parts with better mechanical and machining properties as compared to any other casting process.	[78,86]
Friction stirring process It is a technique that can change the microstructure and mechanical properties through plastic deformation.		(a) It provides low production cost in a short time. (b) It needs a simple and inexpensive setup.	[87,88]

**Table 4:** Comparison between the powder and wire-based LMDs.

Powder-Based LMD		Wire-Based LMD		References
*Pros*	*Cons*	*Pros*	*Cons*	
Various materials can be processed. Complex shapes can be built. Functionally graded materials are manufactured.	It results in high porosity percentage. Material cost is high. Deposition efficiency is less. Powder particles lead to serious health issues.	Material waste is lesser as compared to powder-based LMD. The deposition rate is high. The feedstock is less expensive than powder-based LMD.	The material’s feed rate cannot be controlled. It requires high energy. The dilution rate, in the substrate, of the deposited material is higher than powder-based LMD.	[93,94,95,96,97]

**Table 5:** Integration of powder- and wire-based LMDs.

Powder + Wire-Based LMDs		References
*Pros*	*Cons*	
Higher deposition efficiency. Higher energy valorization. Optimum resource consumption.	Difficulty in the integration of powder- and wired-based LMDs. Staff training, evaluation and testing of produced parts will cause a cost increment.	[92]

**Table 6:** Continuous and discontinuous TMCs formation techniques.

Continuous Reinforced TMCs Formation Techniques			
*Technique and illustration*	*Pros*	*Cons*	References
Lay-up methodology It involves alternating depositions: Woven fiber rugs + 0.90–0.16 mm thick Ti-alloy foils.	It can accommodate high and more extended fiber contents in comparison with the spray technique. The usage of low-temperature resins results in low tooling cost.	It uses excessive foil and fibers. It results in homogenous fiber dispersion and fabrication difficulties.	[105]
Induction plasma deposition It uses an inductive high-frequency plasma to melt and sprinkle the fine-grained microstructures of Ti-matrix onto a wound fiber drum.	The fiber spacing is highly maintained. Any metal matrix, which can be transformed into powder, is used in this process.	A strict composition control is needed.	[106]
Physical vapor accumulation It comprises the build-up of matrix onto a solitary fiber layer via vaporization of a metal matrix using an electron beam or magnetron sputtering.	The distribution of fiber is excellent. The complex shapes can be produced.	The production cost is high. Specialized skills are required. It is relatively a slow process.	[107]
Tape casting It contains slurry formation; then, the reinforcements are coated with slurry and cut into desired shapes.	It requires consolidation steps to obtain homogenous material, which increases the production cost.	The contamination control is difficult due to titanium reactive nature and the presence of polymeric additives.	[108]
**Discontinuous Reinforced TMCs Formation Techniques**			
Powder metallurgy It involves the homogenous mixing of various powders/halide salts to produce ex-and in-situ TMCs	A most suitable technique to produce TMCs. The production cost is reasonable. The production rate is high.	Homogenous mixing is a critical step. Sometimes the surface coating is needed to reach homogenous properties. The selection of reinforcement presents a critical role in TMCs’ final features.	[109,110]
Rapid solidification process In this process, “atomization” technique is used; reinforcements are added into the molten material. It is mostly used for in-situ TMCs.	It is a straightforward technique. The investment cost is lesser than the other techniques.	It requires high heat as an input. A difference in the reinforcements and matrix densities results in the MMCs with non-uniform properties. The processing temperature can affect the size and scale of particulates.	[111,112,113]

**Table 7:** Summary of MMCs mechanical properties.

Study by	MMCs by LMD	Hardness (HV)	Ultimate Tensile Strength (MPa)	Yield Strength (MPa)	Elongation (%)	Wear Loss (µm^2^)	References
Li et al.	WC + Ti-wire	500	-	-	-	-	[141]
Bi et al.	Inconel 625 + TiC particulates (0.25/99.75; 0.50/99.50; 1.0/99.0)	285; 310; 312; 320	840; 930; 980; 990	530; 650; 642; 690	16; 19; 28; 21	-	[142]
Gopagoni et al.	Nickel (80 wt.%) + Titanium (10 wt.%) + Carbon (10 wt.%)	370	-	-	-	-	[143]
Wang et al.	TiC particulates (0; 5; 10; 15; 20; 30 vol.%) + Ti6Al4V	375; 425; 427; 432; 475; 477	1100; 1200; 1100; 1000; 900; 700	-	5; 1; 0.8; 0.7; 0.5; 0.1	-	[56]
Hong et al.	Inconel 718 + TiC (Laser energy = 80; 100; 120; 160 kJ/m)	375; 400; 410; 450	-	-	-	-	[144]
Sateesh et al.	Ni-P + SiC (0; 1; 3; 5 wt.%)	300; 350; 390; 375	800; 900; 910; 300	400; 400; 410; 200	12; 14; 15; 2	-	[145]
Zhang et al.	Ti+TiC (10; 20; 40 vol.%)	260; 301; 336; 503	585; 575; 590; -	520; 520; 515; -	19; 2.5; 1.5; -	309,022.1; 196,579.5; 125,786.7; 107,735.6	[146]
